# Inhibition in the Dynamics of Selective Attention: An Integrative Model for Negative Priming

**DOI:** 10.3389/fpsyg.2012.00491

**Published:** 2012-11-15

**Authors:** Hecke Schrobsdorff, Matthias Ihrke, Jörg Behrendt, Marcus Hasselhorn, J. Michael Herrmann

**Affiliations:** ^1^Bernstein Center for Computational Neuroscience GöttingenGöttingen, Germany; ^2^Institute for Non-Linear Dynamics, Georg-August-Universität GöttingenGöttingen, Germany; ^3^Non-Linear Dynamics, Max-Planck Institute for Dynamics and Self-OrganizationGöttingen, Germany; ^4^Georg-Elias-Müller-Institute, Georg-August-Universität GöttingenGöttingen, Germany; ^5^German Institute for International Educational ResearchFrankfurt/Main, Germany; ^6^School of Informatics, Institute for Perception, Action and Behaviour, The University of EdinburghEdinburgh, UK

**Keywords:** selective attention, computational modeling, negative priming, connectionist models

## Abstract

We introduce a computational model of the negative priming (NP) effect that includes perception, memory, attention, decision making, and action. The model is designed to provide a coherent picture across competing theories of NP. The model is formulated in terms of abstract dynamics for the activations of features, their binding into object entities, their semantic categorization as well as related memories and appropriate reactions. The dynamic variables interact in a connectionist network which is shown to be adaptable to a variety of experimental paradigms. We find that selective attention can be modeled by means of inhibitory processes and by a threshold dynamics. From the necessity of quantifying the experimental paradigms, we conclude that the specificity of the experimental paradigm must be taken into account when predicting the nature of the NP effect.

## 1 Introduction

Selective attention enables goal-directed behavior despite the large amount of ongoing input to the sensory system. This ability is strongly linked to the problem of how information is ignored. Contradicting an earlier understanding that active attention to some objects requires passively ignoring others, an experiment by Dalrymple-Alford and Budayr (1966) revealed, in a series of Stroop tasks an active nature of the suppression of irrelevant stimuli. While the original Stroop (or Jaensch) test did not use a systematic repetition of color and color words, here the stimulus cards were designed such that the ignored meaning of a color word became the color of the next word shown. This led to slower responses as compared to unrelated stimulus colors. Even if the semantic meaning of the words had been ignored, it must have entered the cognitive system to produce the characteristic interference.

Since then, several standard negative priming (NP) paradigms have emerged featuring various dimensions in which priming can occur, e.g., the identity of stimulus objects (Fox, 1995) or their location on the display (Milliken et al., 1994). The stimulus set has also been varied, e.g., pictures (Tipper and Cranston, 1985), shapes (DeSchepper and Treisman, 1996), words (Grison and Strayer, 2001), letters (Frings and Wühr, 2007), sounds (Mayr and Buchner, 2007), or colored dots (Neill, 1977). All paradigms have in common, stimuli containing targets that are to be attended and distractors that are to be ignored. Experimental conditions depend on Stimulus repetitions, particularly the role of a repeated object as target or distractor in two successive trials. Variations of this basic setting include the manipulation of experimental parameters like the time between two related trials, the number of distractors, and the saliency of the distractor. The sometimes contradictory results of such variations will be considered in more detail in Section 2.3. Because of the controversial nature of the NP effect, a variety of interpretations have been developed, but so far none of the theories is able to explain all aspects of the effect. Various underlying mechanisms have been proposed to act at different stages of the processing of the stimuli each justified by a certain experimental result. The theories also diverge with respect to the basis of the effect, i.e., whether it is a memory phenomenon or an effect of attention. They all agree, however, on the critical role of temporal processing for an understanding of NP.

We are particularly interested in the neurophysiological mechanisms behind attention and ignoring of perceptual information. Attention is, in principle, a form of guidance of neural activity toward relevant resources. If ignoring of stimuli or stimulus features is an active process, then those resources are subject to suppressive effects of some kind. In principle, these could be maintained by various processes, e.g., elevated thresholds, synaptic depression, or competition involving homeostatic plasticity. However, considering that attention is essentially guided by processes in the prefrontal cortex and the fact that prefrontal feedback is typically given by inhibitory signals (Knight et al., 1999), it seems likely that inhibition plays a key role in the effects of selective attention.

In the model presented here, inhibition serves multiple functions: it not only underlies attention by suppressing irrelevant stimulus components, but is essential in the formation of bound states that represent objects as synchronized set of feature-related activity and is assumed to underlie the selection of action. Corresponding to the multiple uses, inhibition occurs in several forms. At the sensory level, inhibition is merely a relative advantage of one of the perceived features that is initiated by top-down input. In this case, the model is ignorant to the particular form of suppression, which can be implemented in different but mathematically equivalent forms, e.g., as an adaptive threshold. This indifference is due to the generality of our approach and allows us to express several conflicting theories from the psychological literature by the same formal model component.

In the feature binding component of our model inhibition occurs in a uniquely defined form: object-encoding activations in the binding layer are stabilized by lateral inhibition. Although here also alternatives are mathematically possible, there is no psychological or neurophysiological evidence for a fine-tuned mechanism as proposed by Schrobsdorff et al. (2007a). Finally, inhibition is realized in a more schematic form in action selection which we have included in the model in a form analogous to the perceptual or frontal modules rather than as a realistic representation of the motor system.

A further main contribution of the present study is a single and comprehensive computational model, combining the different theories such that it is able to express the behavior predicted by each of the NP theories^1^. To deal with apparent inconsistencies and incompatibilities across the theories, we employ two strategies. First, we choose a dynamical formulation, whose natural mathematical form, allows us to identify similarities that are not obvious from the theoretical conclusions of specific experiments, and whose structure can be directly related to physiological evidence of cognition. Second, we will use a set of configuration parameters that function as weights or semaphores and can scale-down or switch-off a component that is not postulated in a certain theoretical context. In other words, all the model components can work together but often such preselected subsets of components are sufficient to describe a given empirically developed theory. It is crucial to remark that the different roles of inhibition are always present in the variants of the model that are implied by the literature, except for the retrieval module which is not discussed in some accounts. Also generally, the choice of the configuration is unambiguously specified by the psychological account in all major theories of NP. In the present formulation of the general model for negative priming (GMNP) there are seven optional components, but extensions are easily possible, should newer experimental evidence imply additional contributions to the NP effect.

We will describe in detail how a computational model can be constructed along these lines that comprises all potentially relevant processing stages for an NP task. The result is not only a comprehensive model of the theories of NP, but more generally, a framework for perception-based action in natural or artificial cognitive systems. The system is explicit in the sense that the components are mathematically defined. The system is also connectionist, i.e., the interaction between the components represent the task (see Figure 3) which is realized either by design or in the wider context by a learning process. Finally, the system is dynamic, i.e., the activity levels of all components change in time and excite, inhibit or modulate each other. This reflects the importance of the time course in NP as well as in general behavioral contexts.

The paper is organized in the following way. We will first clarify terminology, deepen the discussion on how to concretize psychological theories, present the NP effect, give an overview on the biological background of the model units and finally explain how these enter into the proposed GMNP. The second section thoroughly reviews existing theories of NP. Specifically, we give a historical overview of the development of theories and what additional conclusions were drawn in experimental papers. The quantification of theories and how they are integrated in the framework of the GMNP is followed by a technical chapter that describes the implementation of the model in a way allowing researchers to reproduce the simulations. Finally the behavior of the GMNP in various NP paradigms is shown. The concluding discussion summarizes these results and considers the potential of the model beyond the described target application in NP.

## 2 Materials and Methods

We present an integrative connectionist model of NP. For a thorough description of the model and the necessity of its parts, this section is organized as follows. After defining basic experimental nomenclature we very briefly present a generic NP experiment to introduce the viewpoint of NP research. Next, we summarize the various and diverse modulations of NP when faced with a wide range of experimental variations, thereby showing the sensitivity of the phenomenon and thus the requirement of a rather complex model. Then, we review a number of theoretical accounts that were postulated to explain a certain aspect of NP. Those theories will be incorporated in our model. After an overview of the GMNP, we describe the role of the individual model components in detail, and finally, the rigorous mathematical formulation of the GMNP is presented.

### 2.1 Definitions

In the present study we will use the following definition: NP is a slowdown in reaction time in a repetition condition where a former distractor has become target. Because we define the term NP by reaction time differences, we shall not use it to denote the ignored repetition condition. Instead we will label the condition by two (or four) letters that indicate the configuration of stimuli in a trial consisting of a prime and a probe display (see Christie and Klein, 2001). Generally, the first letter contains information about which part of the prime display is repeated in the probe display: the letter D represents the distractor, while T represents the target. The second letter indicates the role the particular object has in the probe display. For example, the string DT refers to the condition in which the prime distractor (first letter D) is repeated in the probe trial as a target (second letter T), which denotes the traditional NP condition. If no stimulus is repeated, the condition is denoted by CO. In case both objects are repeated there is a second pair of letters appended for the second object. Because a target and a distractor are each shown in the prime and the probe display, seven relevant combinations of target-distractor relations are possible, see Table 1.

**Table 1:** **The priming conditions of a paradigm with one target and one distractor in each of the prime and probe display**.

	Prime display		Probe display		
	Target	Distractor	Target	Distractor	
TT	A	B	A	C	Target(*n* + 1) = target(*n*)
DT	A	B	B	C	Target(*n* + 1) = distractor(*n*)
TD	A	B	C	A	Distractor(*n* + 1) = target(*n*)
DD	A	B	C	B	Distractor(*n* + 1) = distractor(*n*)
DDTT	A	B	A	B	Target and distractor are repeated
DTTD	A	B	B	A	Target and distractor are swapped
CO	A	B	C	D	Two new stimuli

### 2.2 A negative priming experiment

**Figure 1:**
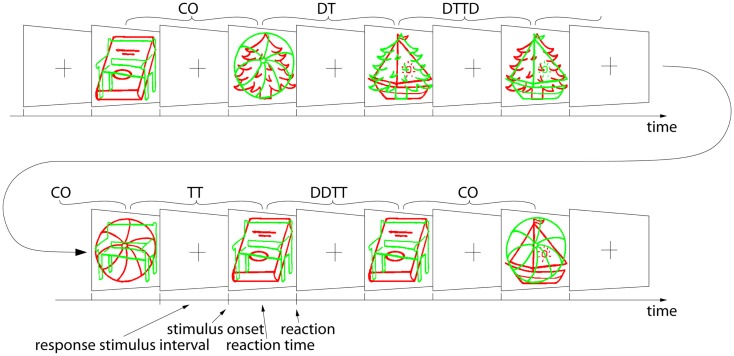
**Example of a sequence of stimuli**. Consecutive screens are shown. Either stimuli or a blank screen followed by a fixation cross is displayed. Acronyms are explained in Table 1.

We will now very briefly discuss a prototype NP experiment that we will refer to in the following discussion. The experiment has been adapted from the classic study by Tipper (1985) and is presented in detail in Schrobsdorff et al. (2007b). Subjects are instructed to name the green pictogram as quickly and accurately as possible (see Figure 1). Stimuli are six different objects, represented by hand-drawn pictograms that are either shown in green or in red. We use voice recording together with a sound level threshold to determine the reaction time for every trial. As the experiment is run in German, possible responses are German names of simple objects that begin with a plosive and consist of a single syllable: *Baum* (tree), *Bus* (bus), *Ball* (ball), *Buch* (book), *Bett* (bed), and *Bank* (bench), for a sharp, and thus easily detectable onset of the sound signal. For efficiency reasons, we present the trials continuously, such that every trial primes the subject for the following trial (see Ihrke and Behrendt, 2011, for a discussion of the implications of this procedure). Object presentation is balanced in the different priming conditions as well as in their appearance as target and distractor. Implemented priming conditions include CO, DT, TT, DDTT, and DTTD, see Table 1 and Figure 1.

A stimulus display consists of two overlapping line drawings, a green target, and a red distractor object. The subject is instructed to name the target objects aloud and ignore the superimposed red objects. They were told to answer as quickly and as accurately as possible. Then, after a blank screen period and the presentation of a fixation cross, the next display is presented. Mean reaction times of the different priming conditions, the standard deviations, and the effect strengths, i.e., the difference to CO trials, are shown in Table 2. For details, see Schrobsdorff (2009). DTTD trials produce the slowest responses, followed by DT and CO trials, whereas the responses to TT trials are faster than control and DDTT trials produce the fastest responses.

**Table 2:** **Reaction times, standard deviation, and priming effects, i.e., the differences of control (CO) reaction time and reaction time of the according condition (DT, DTTD, TT, TDDT)**.

	〈RT〉 (ms) (SD)	Effect (ms)
CO	660.22 (62.85)	–
DT	681.57 (69.65)	−21.36
DTTD	685.92 (78.04)	−25.70
TT	625.02 (65.29)	35.20
DDTT	600.69 (70.56)	59.53

The experiment shows how the repetition of stimuli can influence reaction times in a NP paradigm. A repetition of relevant stimuli leads to prominent speedups (TT, DDTT conditions), whereas a presentation of formerly irrelevant stimuli as the current target results in slowed reaction times (DT and DTTD conditions) as compared to the control condition.

### 2.3 Characteristics of the negative priming effect

**Figure 2:**
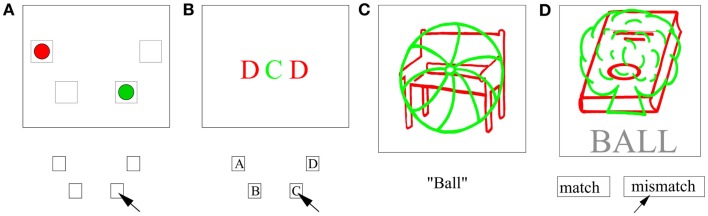
**Four different paradigms for NP**. **(A)** The location priming paradigm reveals NP in the encoding of space. **(B)** The flanker task implements a stimulus response mapping. **(C)** Responses are given as vocalization in the voicekey paradigm. **(D)** The word-picture comparison paradigm has the advantage of a disentanglement of target identity and response. The examples have been adapted such that green always defines the target.

Negative priming has been found in a wide variety of experimental contexts (for reviews, see Fox, 1995; May et al., 1995; Tipper, 2001; Mayr and Buchner, 2007). For example, NP has been elicited using different stimuli such as line drawings (Tipper and Cranston, 1985), letters (Neill and Valdes, 1992; Neill et al., 1992), words (Grison and Strayer, 2001), auditory stimuli (Banks et al., 1995; Buchner and Steffens, 2001; Mayr and Buchner, 2006), and nonsense shapes (DeSchepper and Treisman, 1996). NP has been found in various tasks including naming (Tipper, 1985), same-different matching (DeSchepper and Treisman, 1996), Stroop-like tasks (Neill, 1977), and spatial localization (Milliken et al., 1994; Park and Kanwisher, 1994; May et al., 1995; Kabisch, 2003), see Figure 2 for four example paradigms.

The NP effect is sensitive to a large number of parameters. Most paradigms show a particular aspect of NP, but no global pattern of results exists (Fox, 1995). It has been shown that NP can depend on the length of the response stimulus interval (RSI) between prime and probe (Neill et al., 1992; Kabisch, 2003; Frings and Eder, 2009). However, there are also studies reporting a constant NP effect for varied RSIs (Hasher et al., 1991, 1996; Tipper et al., 1991). Surprisingly, for very short RSIs, a DT condition can produce a facilitatory (Lowe, 1985), or hampering effect (Frings and Wühr, 2007). At the other extreme, an experiment revealed NP after a month using nonsense shapes which are very unlikely to be seen in other circumstances (DeSchepper and Treisman, 1996). For continuous presentation of trials, the proportion of preprime RSI and current RSI influences NP (Neill and Valdes, 1992; Mayr and Buchner, 2006), but not reliably (Hasher et al., 1996; Conway, 1999). In the absence of distractors in the probe trial during a DT condition, NP vanishes or even reverses to facilitation (Allport et al., 1985; Lowe, 1985; Tipper and Cranston, 1985; Moore, 1994). A more salient prime distractor increases the magnitude of the NP effect in DT conditions (Grison and Strayer, 2001; Tipper, 2001). NP is reduced or even reversed to facilitation when the emphasis is put on speed rather than accuracy (Neumann and Deschepper, 1992). Increasing the perceptual load, e.g., by raising the number of distractors presented in a single trial, leads to less NP (Lavie et al., 2004). In other settings a higher number of prime distractors causes an increase of NP (Neumann and Deschepper, 1992; Fox, 1995). The inclusion of TT trials or single target trials in the presentation sequence enhances NP (Neill and Westberry, 1987; Titz et al., 2008). A short presentation time of prime and probe stimuli attenuates NP (Gibbons and Rammsayer, 2004). NP vanishes if the target is presented a bit earlier than the distractor in the prime trial. On the other hand, if the prime distractor is shown simultaneously with the prime target but blanked after a short time, NP is observed (Moore, 1994). If the prime display contains a single stimulus that is masked, subjects reporting awareness of the prime object show positive priming, while subjects not aware of the object show a NP effect (Wentura and Frings, 2005). In subliminally primed trials the presence of a distractor in the probe leads to negative priming, whereas the absence of a probe distractor leads to a positive priming effect (Neill and Kahan, 1999).

### 2.4 Theories of negative priming

Because of the sensitivity of the NP effect to numerous factors, a variety of theories have been proposed to explain the disparate experimental facts. None of the present theoretical descriptions, however, explains all observation related to the NP effect, cf. Section 2.3. In the present section we will give an overview on the most relevant approaches.

#### 2.4.1 Distractor inhibition theory

In the first attempt to explain NP, the inhibition hypothesis (Neill, 1977; Neill et al., 1990) inhibition plays a central role. Later, this hypothesis branched into distractor inhibition theory (Tipper, 1985, 2001; Tipper and Baylis, 1987; Tipper et al., 1988, 1991, 2002; Tipper and McLaren, 1990; Houghton and Tipper, 1994, 1996), and episodic-retrieval theory (Neill and Valdes, 1992, see Section 2.4.2).

In the distractor inhibition theory, inhibition is complemented by an attentional selection process, i.e., the direct feed-forward excitation induced by the (visually) perceived stimuli. The slowdown of the reaction in the probe trial can be understood as a direct indicator of the amount of distractor activation in the prime display. Persisting inhibition is assumed to drive the distractor representation below a baseline activation after stimulus offset. Selection is said to operate on a semantic or postcategorial level (Houghton and Tipper, 1994). It therefore also explains findings that report NP in semantic priming tasks (Tipper and Driver, 1988).

The NP effect increases with growing saliency of the distractor (Lavie and Fox, 2000; Grison and Strayer, 2001; Tipper et al., 2002). This effect can be very well explained in terms of the inhibition model, since a stronger distractor would require more inhibition, causing a stronger inhibitory rebound, and thus leading to a more prolonged reaction time. Distractor inhibition theory can explain the larger NP effect by a stronger activation and thus more inhibition for distractors (Craik and Lockhart, 1972; Craik, 2002). Therefore, more deeply processed stimuli produce larger NP effects.

Opposingly, distractor inhibition theory fails to explain the experimentally observed dependency of NP on the RSI: if the representation of a distractor object is inhibited, the impact of inhibition should be strongest immediately after the selection, because the inhibition is assumed to decay with time. Although there is a general trend of NP to decay with increasing time between prime and probe (Neill and Valdes, 1992), no NP is observed in several studies when the RSI is very short or non-existent (Lowe, 1985; Houghton et al., 1996).

#### 2.4.2 Episodic-retrieval theory

Proposed by Neill and Valdes (1992), episodic-retrieval theory supposes that if a task is executed over and over again, memories of past trials are more and more used in the current trial. NP is then assumed to be the result of automatic retrieval of the prime episode during probe processing causing a hampering interference. It is argued that the retrieval is triggered by the similarity of prime and probe episodes. As the information from the retrieved episode in a DT trial is inconsistent with the current role of the repeated object as a target, retrieved and perceived information are in conflict. Resolving the conflict is time consuming and results in the slowdown of the reaction time.

According to later extensions by Neill (1997), the main determinants of the strength of retrieval are the recency of the memory trace and the strength of the memory representation of the former trial. Recency as a relevant factor receives empirical support from studies that show a negative correlation between RSI and NP effect (Neill and Valdes, 1992).

A facilitated response at very short RSIs (Lowe, 1985) is difficult to explain in terms of the episodic-retrieval framework. Another weakness of this approach is the empirically found effect of semantic NP (e.g., Waszak et al., 2005): the absence of perceptual similarity should prevent any retrieval to occur thus predicting the absence of any priming effects.

#### 2.4.3 Response-retrieval theory

A relatively recent version of the episodic-retrieval theory focuses on the encoding and retrieval of processing operations that have been carried out during trial processing – in particular the response (Rothermund et al., 2005). The theory builds on results from the research on event-files (Hommel, 1998, 2004, 2005), which investigates the encoding and retrieval of perception-action bindings. Since the retrieved response conflicts with the response required by the task in DT trials when a naming task is implemented, NP is explained as an interference between the retrieved and the currently required response. One particular merit of this response-retrieval theory is therefore that it points to the inherent confounding of the priming condition and the response relation in most NP paradigms: usually DT trials are accompanied by a response switch, whereas TT trials require the same response. The response-retrieval approach postulates that every reaction time difference in priming paradigms is explained by the retrieval of a past response depending on the perceptual similarity between the two displays. In their initial study, a letter-matching task initially developed by Neill et al. (1990) was adapted in order to orthogonally vary repetition or non-repetition of the response and priming conditions (Rothermund et al., 2005). Since the proposition of response-retrieval theory, many studies have found empirical support for it (e.g., Mayr and Buchner, 2006; Ihrke et al., 2011).

#### 2.4.4 Temporal discrimination theory

Temporal discrimination assumes a classification of stimuli as *old*, where a response can be retrieved from memory, or *new*, where a response has to be generated from scratch (Milliken et al., 1998). The classification consumes time depending non-monotonically on the similarity between the current stimulus and a memory trace: the classification as *new* is fast when prime and probe stimuli are very dissimilar. The classification as *old* is fast when the displays are identical. Intermediate similarities, however, such as in DT trials where the prime distractor is repeated but not in the same color, the decision whether the display is *old* or *new* takes longer (see also Neill and Kahan, 1999; Healy and Burt, 2003). Hence, both NP and positive priming effects can be explained with this mechanism.

Temporal discrimination and episodic-retrieval theories are quite similar in structure. Most criticism toward temporal discrimination relies on the equivalence of processing time after the *old*/*new*-classification. Temporal discrimination tacitly assumes that the direct computation of a response is completely different from a retrieval of the answer from memory. Thus no statement exists that these processes take an equal amount of time. Another weak point of temporal discrimination theory is the assumption that classification and retrieval or direct generation of a response is processed serially. Most processes in the brain work in parallel, and therefore a simultaneous computation (at least partly) of the *old*/*new* signal together with a directly computed answer and the retrieval of past episodes is more plausible.

#### 2.4.5 Dual mechanism theory

Since there is evidence in support of both inhibitory and episodic-retrieval processes, several authors have proposed that both mechanisms should be active. This notion has been termed dual mechanism theory. Originally, May et al. (1995) proposed that inhibition as well as memory retrieval can be the source of NP and the experimental context specifies which of the two mechanisms is expected to operate. Tipper (2001) argued that it is important to note that distractor inhibition and episodic-retrieval theories are not mutually exclusive, and both inhibitory and retrieval processes could be involved in the emergence of NP. Although retrieval processes can be responsible for producing NP effects, inhibitory processes are still required in selecting information for goal-directed behavior. In most tasks, NP will supposedly be caused by a mixture of contributions from persisting inhibition and interference from retrieval. Because these processes may sometimes oppose each other, it is difficult to distinguish them by means of behavioral measures like reaction times and error rates (Gibbons, 2006). However, depending on the context and other experimental factors, the contributions of inhibitory and retrieval processes might vary considerably (Kane et al., 1997; Tipper, 2001). Nevertheless, Gamboz et al. (2002) revealed in a meta-analysis that there is no significant evidence for a paradigm to produce patterns of results favoring either inhibition or retrieval theories, pointing to simultaneous presence of inhibition and retrieval. Such a conclusion supports the general framework adopted in the GMNP, presented in this paper.

#### 2.4.6 Global threshold theory

Kabisch (2003) developed the imago-semantic action model (ISAM) with the hypothesis of a threshold variable whose value decides to which items the system will respond from perceptual input. The threshold adapts according to the current average activation of representations of objects. Additionally, a forced decay of activation is assumed in the model if residual activity is partly overwritten by perceptual input of a new stimulus. The ISAM can account for positive as well as NP as shown by computer simulations (Schrobsdorff et al., 2007b). It differs from distractor inhibition theory (Section 2.4.1) by postulating only facilitative input and passive decay in the absence of input.

The ISAM gives a comprehensive account of action selection. The presented objects are assumed to undergo pre-attentive processing and a perception stage, resulting in an abstract cognitive representation of the objects. Formally, the decision between target and distractor is determined by the task instruction, which is made accessible to the model via a semantic feedback loop. In contrast to the early visual processes, the decision is guided by attention and a conscious application of the task instruction. The semantic object representations are assumed to be initially processed automatically according to a relevance rating based on low-level features such as motion or color. If more than one or no option for suprathreshold actions exist, the threshold adapts until only one option remains. The relative relevance of stimuli can be affected in a posterior rating. According to the dual-code hypothesis of Krause et al. (1997), assigning modified relevance values to the object representation happens in a semantic space. The activation corresponding to a target is further amplified by a top-down feedback loop informed of the task, such that even if low-level perceptual features result in a higher input to the distractor, the target representation eventually becomes significantly stronger than that of the distractor.

### 2.5 A general model for negative priming

The existing theories of NP have pointed to several mechanisms that are likely to play a role in producing NP. However, it is very important to keep in mind that fundamental research in psychology uses statistical properties of experimental data in order to interpret human behavior. On the one hand, behavioral experiments tend to produce largely varying results which reflect the complexity of the involved systems and the sensitivity of the effect. On the other hand, the interpretation of results is usually not unambiguous. Both aspects provide a base for the arduous and controversial discourse that is necessary for a clarification of the psychological phenomenon.

#### 2.5.1 Computational modeling of negative priming

Theories explaining NP can be categorized roughly into memory-based and activation-based approaches. The first group assumes the memorization of a trial and eventually a retrieval of the information in the next trial. The latter group assumes NP to be caused by interference of trial processing with persistent activation from former trials. Within both groups a number of variants were produced, many of which were created to explain a specific pattern of results. Comparability is nevertheless an issue that calls for a more comprehensive approach.

It seems reasonable to focus on the interaction of underlying processes rather than on *ad hoc* definition of data features. However, a substantial reduction of complexity is already achieved by the careful design of experiments and all theoretical explanations are based on the assumption that the complexity of experimental data can be further reduced by identifying repeating patterns in the data. A crucial point in the specification of mechanisms producing NP seems to be the exact time course of processing in a trial where a previously ignored stimulus has to be attended in comparison with the processing of an unprimed stimulus.

In order to tackle the diverse paradigms and the incomparability of the theories, we designed a computational framework for perception-based action selection in the NP paradigm by means of physiologically justified building blocks, each showing biologically plausible dynamics. The general architecture is a dynamical implementation and generalization of the model studied in Hommel (2004). The simple thresholding mechanism responsible for the creation of perception-action bindings in Hommel’s model is generalized using dynamic and weighted bindings. The obtained implementation inherits freedom of interpretation from the underlying theory. Additionally, the implementation adds further degrees of freedom by the introduction of a number of technically implied parameters. The benefits of an implementation are, nevertheless, obvious. The computational model reduces the risk of misinterpretation if the source code is available to other research groups for an independent reproduction of the results.

In order to reproduce observed results, most models have to undergo a precise fitting of model parameters, which is often a very subjective process. Therefore, great care has to be taken of the distinction between results due to parameter fits and predictions generated by the internal dynamics of the model without further fitting. A different way to benefit from a computational model is to analyze the structural result after fitting, which carries a formalized version of the fitted data. We build a computational model comprising most of the mechanisms suspected to play a role in the neural processing in NP. The outcome is not only a meta-model for NP, termed GMNP, but in itself a simplified model of the brain as a framework for action selection based on perception. We addressed the tradeoff between biological realism and understandability by implementing all mechanisms as separate blocks keeping the internal dynamics simple by implementing the exponential dynamics previously developed in Schrobsdorff et al. (2007b).

#### 2.5.2 Different paradigms

A common explanation for the divergent results of NP studies is the difference of the conducted experiments. Each paradigm has special aspects concerning trial processing beginning from perceptual pathways up to the response modalities. Differences in the task are assumed to affect the involvement of memory and inhibitory modulations. Thus it is important to build a GMNP that is flexible enough to evaluate a variety of paradigms, i.e., not only to computationally reproduce interesting priming experiments, but also to quantify the difference of paradigms. Such a formulation contributes directly to the clarification of the debate about the influences of experimental design on NP. Most importantly, the model has to accept different stimuli and to produce distinct forms of responses. In addition, a mechanism formalizing the actual task for a paradigm is necessary.

A computational implementation (Houghton and Tipper, 1994) of an artificial neural network qualitatively explains NP by an inhibitory rebound naturally emerging from the network connections between excitatory and inhibitory cells homeostatically balancing the state of a so-called property unit. Perception is assumed to be split into the detection of single features which are bound into object representations by hardwired connections. The model has a very general connection scheme to be able to describe selective attention in a variety of situations.

This connectionist implementation of distractor inhibition theory is designed to deal with diverse perceptual inputs. Stimuli are decomposed into their features and recognized by specialized feature units. Then the object identity is realized by a flexible feature binding mechanism (Treisman, 1996). The GMNP implements a binding mechanism for feature representations by means of persistent spiking activity (Schrobsdorff et al., 2007a) that is similar to the abstraction of population activity in a neural network leading to the exponential dynamics (Section 2.7.1). Different response modalities are included in two separate layers for semantic representations and response actions. Between the two layers, a central executive implements a mapping to account for different tasks (e.g., comparison). The central executive also provides information about which feature instance codes for the target and distractor, and which feature dimension is relevant for the response (see Section 2.6.5). Before presenting a formal version of the GMNP (Section 2.7) we will specify the model components based on the discussion above.

### 2.6 Model components

**Figure 3:**
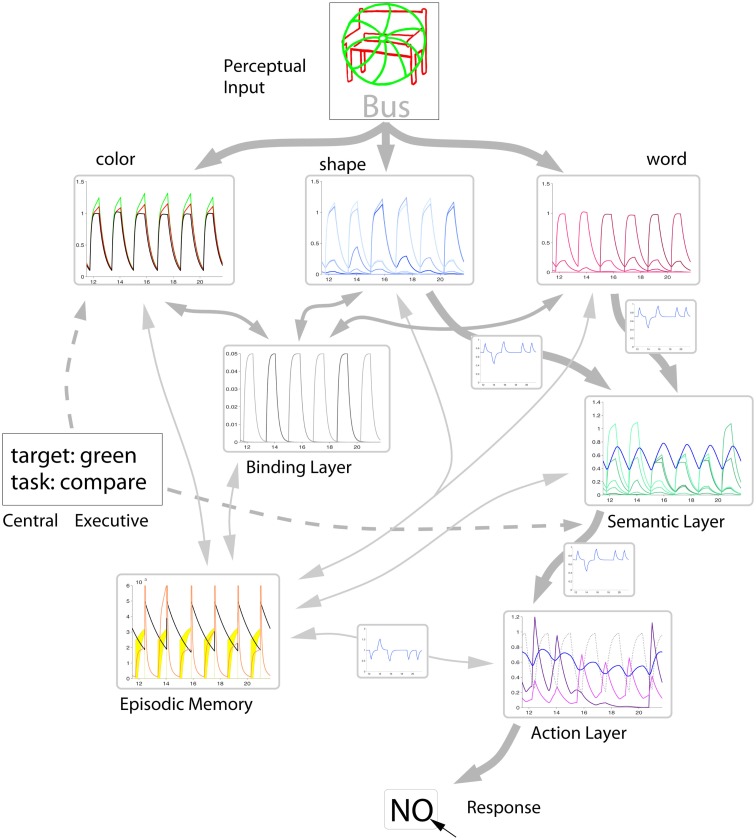
**Interaction scheme of the different components of the GMNP**. Perceived stimuli are decomposed into single features, each of which is represented in a single variable in the corresponding layer. Object identity is maintained by activations in the binding layer, associating the different features of a stimulus object. Most paradigms require a semantic evaluation of the stimuli in order to generate a response. Therefore, the semantic layer gates information flow from the relevant features to the action layer which decides on the action to perform. Parallel to the information flow from perception to action a so-called central executive steers the model behavior with regard to the current task, i.e., providing information about the target and the mapping of semantic variables to actions. According to the similarity of the percept and a memorized stimulus configuration, the memory layer feeds back information of the former trial. The similarity signal also affects the effectiveness of transmission between features, semantic layer and actions as well as between memory itself and actions, the latter inversely to the first.

The GMNP is formulated in a distributed way in which several specialized layers interact according to the flow of information in the brain during perception-based action selection tasks. An overview of the model structure is shown in Figure 3. Information is mostly fed from top (perceptual input) to bottom (action execution), except modulating layers like the binding layer, episodic memory, and the central executive. Perceptual input is fed into various feature layers, each representing a certain aspect of the presented stimuli. The object entity is represented in a feature binding layer which forms a link between all features of one object. Depending on the task, the model implements a mapping of relevant features into a semantic layer, which is equipped with a decision mechanism to sort out the semantic representation relevant for an accurate response to the task. The winning information is passed to the action layer, which chooses between different possible responses on the basis of the available information. Aside from the above pathway, is a memory layer which stores the network state from former episodes and feeds this information back when helpful for a quick response.

#### 2.6.1 Feature layers and feature binding

In the visual pathway the information from the retina is decomposed into low-level features which are represented by different subsets of neurons (Van Essen et al., 1992). Later, the low-level representations are recombined to form higher-order features of objects from visual input (Prinzmetal, 1995). Feature decomposition entails the disadvantage that the distributed information about an object needs to be bound together for the recognition of objects as entities, a concept known as feature binding (Treisman, 1996). The neural implementation of such bindings is still under discussion (Hommel, 2004) but synchronization is likely to play a role (Singer, 1995). In the GMNP, we implement this mechanism in terms of a feature binding model on the basis of localized excitations in a spiking neural network (Schrobsdorff et al., 2007a).

In order to cover the paradigms featuring visual stimuli, we equip the current implementation of the GMNP with feature layers to detect color, shape, location, and word(-shape). A visual stimulus is recognized by particular activation in each of the corresponding feature layers and a binding between them. Binding of the features of a certain object is realized as a set of features, and a binding strength which specifies both the importance of the object to working memory and also the effectiveness of activation exchange between the features of the corresponding object. The GMNP is able to keep a small number of such bindings active at a time.

In the formation of binding, attention seems to form a crucial role, as neuromodulators associated with attention are essential for the formation but not for the maintenance of bindings (Botly and De Rosa, 2007). In terms of the GMNP this means that objects from currently perceived stimuli are bound, and the binding can survive the vanishing of the perceptual input. Bindings are stable against stimulus changes up to the point where the limited resources are in use, i.e., the maximum number of bindings is reached.

#### 2.6.2 Semantic representations

Some NP paradigms require stimulus evaluation on a semantic level, e.g., the word-picture comparison task: the specialized Stroop cards which are the origin of NP research (Dalrymple-Alford and Budayr, 1966); or the naming of pictograms in the experimental paradigm introduced in Section 2.3. Semantic representations are closely related to language processing (Demb et al., 1995), which is distributed over the entire cortex. Despite the distributed nature of semantic processing (Bookheimer, 2002; Devlin et al., 2002), the GMNP includes only one layer holding the strengths of the semantic representation of a given stimulus (similar to the description in Schrobsdorff et al., 2007b). The GMNP also inherits the attention mechanism, i.e., an adaptive threshold relying on activations in the semantic layer. The threshold controls information propagation to the response layer.

#### 2.6.3 Episodic memory

Episodic-retrieval theory, assumes that previously processed stimuli are stored in episodic memory. In most NP paradigms, the memorized sequence of trials is assumed not to extend beyond the directly preceding trial. The interference of memory with behavior is assumed to depend only on the time elapsed and the stimuli encountered in the meantime. We prefer naming the memory processes relevant in NP as *episodic memory*.

Physiologically, memory encoding is related to activity in the left prefrontal cortex, whereas retrieval is more associated with right prefrontal cortex (Tulving et al., 1994; Fletcher et al., 1997). This is conjectured to be due to different control mechanisms on the two tasks (Craik, 2002). We solve the stability-plasticity problem that memories have to be formed reliably and instantly but have to persist for some time even in the presence of interfering input (Norman et al., 2005; Suzuki, 2006), by implementing a limited number of memory slots that hold the entire state of the system at a certain point in time. Such a memory is assigned a strength which decays with time. Individual instances are the only forms of experience that are represented neurologically, as (Logan, 1988) postulates.

#### 2.6.4 Memory retrieval

Memory research distinguishes between involuntary retrieval and voluntary recollection (Yonelinas, 2002). The so-called familiarity signal is physiologically measurable, and becomes visible in the EEG 300 ms after stimulus onset. Familiarity is assumed to trigger further retrieval, as a spontaneous recognition can lead to recollection (Zimmer et al., 2006; Ecker et al., 2007). Context monitoring means the evaluation of the appropriateness of a retrieved episode (Egner and Hirsch, 2005). Topography, latency, and polarity of the familiarity signal in EEG-data bears resemblance to the *old*/*new* effect related to episodic memory retrieval (Rugg and Nagy, 1989).

The two approaches, episodic retrieval and temporal discrimination theory, predict differing mechanisms controlling the strength of memory retrieval. The first theory assumes that involuntary retrieval is positively correlated with perceptual similarity of the two trials. The latter postulates another perception-based classification of the encountered episode as *old* or *new*. When significant evidence for an old stimulus display is accumulated, full retrieval is triggered, while simultaneously suppressing the direct response generation.

The GMNP performs the computation of a familiarity signal by comparing the current percept with the memorized one. Depending on model parameters emphasizing either episodic-retrieval theory or temporal discrimination, this familiarity can influence further processing in two ways. First, the strength of retrieval can be determined directly, i.e., familiar stimuli cause stronger retrieval-related activity, while unfamiliar stimuli still produce a positive activity. Secondly, the system holds a template time course of a familiarity signal separating the time courses of the familiarity signal while encountering a perfect match of stimulus displays and a pair of subsequent displays that vary in a single feature. Greater familiarity indicates an identical stimulus configuration, while lower familiarity is considered as being produced by a new display. The uncertainty of the signal early in the trial is implemented by the GMNP by a shrinking margin around a template familiarity curve for a nearly identical stimulus, in which the evidence of the display being *old* or *new* is not yet significant.

#### 2.6.5 Central executive

The GMNP aims at a compromise of evidence-based complexity and computational simplicity. Instead of providing mechanisms for the adaptation to different paradigms, we rather map the paradigms to appropriate parameter configurations. The corresponding component of the GMNP is called the central executive (Cowan, 1988) and is understood as an emergent property of interacting subsystems (Barnard, 1985; Teasdale and Barnard, 1993; Bressler and Kelso, 2001). Even if there is no consensus on the necessity of a central executive in memory functions (Baddeley, 1998; Johnson, 2007), we will use the term in order to describe the sudden change in system behavior if it is presented a new task. In this way the GMNP receives information about the task demands, i.e., about a specific paradigm, including the top-down input modulating target or distractor activation and mappings describing the determination of the input to the action layer.

#### 2.6.6 Representing theories of negative priming

The comparison of the different theoretical approaches is one of the major reasons for the design of the GMNP. In order to be able to directly compare the respective impact of each mechanism, the main components of each theory need to be precisely formulated within a common language. In the following, we outline how each of the theoretical approaches is realized in the GMNP.

Distractor inhibition theory is expressed in a straightforward way. The distractor object, i.e., the feature that specifies the distractor, is subject to inhibition. Simultaneously, dynamic activations below baseline are included to model the inhibitory rebound (this constitutes a deviation from the model developed in Schrobsdorff et al., 2007b). Correspondingly, inhibition in the semantic layer is indirectly achieved via the binding between feature and semantic layers.

Episodic-retrieval theory requires explicit modeling of memory and retrieval processes. Therefore, we included short-term memory by adding a dedicated layer that is able to store a snapshot of the state of the dynamic system and that is subject to decay over time. This memory layer is also capable of computing the strength of retrieval determined by the similarity of the current percept and the memory content. Retrieval is modeled by partially restoring former system variables. Memory is updated at the most prominent point in a trial, i.e., when the decision takes place. Response retrieval manifests itself in the GMNP as a simplification of episodic retrieval. Only the system variables of the action layer are restored during retrieval. The retrieval strength is still determined by the similarity of current and stored percept.

Temporal discrimination theory acts on the same episodic memory layer as episodic retrieval. The probability that a stimulus display was just presented can be computed by looking at the similarity between current and memorized percept as described above. This value is highest when both configurations match exactly. The similarity slowly rises from zero to its final value. The current similarity is compared to a prototype similarity signal in order to determine whether the current percept is old or new. In order to be robust against initial fluctuations in the similarity stemming from residual activation of the last trial, the computed difference has to surpass a threshold that is large at trial onset but shrinks with time. If a display is rather similar to the memorized one, the similarity value will stay within the uncertainty interval the longest, preventing an old–new-classification. When the classification is accomplished, temporal discrimination theory assumes the information flow to be affected: in the presence of new stimuli, retrieval is blocked, and direct computation is facilitated. For old stimuli the direct computation is dropped and retrieval will be performed. This is included in the GMNP in terms of a modulation of the transmission strengths between the corresponding layers: from semantic to action for direct computation and from episodic memory to action layer for retrieval.

The spirit of the dual mechanism hypothesis is inherent to the GMNP, because it accounts for all theories at once. By tuning the model parameters, the behavior predicted by each theory can be generated. According to the above discussion it is evident that the mechanisms postulated by inhibition and threshold theory are located in the more sensory part of the system whereas retrieval, even though affecting the entire system, only becomes apparent in later parts, i.e., in the semantic and action layer. As the two mechanisms are implemented at distinct parts of the GMNP, coexistence of the mechanisms is achieved trivially.

### 2.7 Model dynamics

After the examination of the processes involved in an NP task in the previous section, we will now mathematically describe the model. The level of description results from a compromise between the explicitness of the formulas and the complexity of the full system. The basic architecture of the model is simple. Perceptual input enters the system in the feature layers, which passes information to the semantic and action layer. Finally, we describe the behavior of the memory variables.

Activations of feature and object representations follow an exponential fixed-point dynamics (Schrobsdorff et al., 2007b), i.e., the difference of a state variable and a given fixed-point determines the change of that variable while the rate of change is governed by a time constant. This dynamics can be derived from firing rate considerations of a network of spiking neurons, as we show in the following section.

The model has a number of meta-parameters that act as *weights* or “setscrews” (see Section 3.1). In this way the model represents the particular assumptions in each of the theories in Section 2.4. We will not consider a graded likelihood of the assumptions and therefore choose the weights to be either 1 or 0. In this way the GMNP yields quantitative comparisons between the theoretical accounts while continuous weights would result in new theories.

#### 2.7.1 Determining a simple intrinsic dynamics

For the GMNP, we will subsume the mental representation of each cognitive object, e.g., a perceived feature or a semantic category, under a single variable which corresponds neurophysiologically to the activation level in an assembly of neurons. The firing behavior of this assembly is driven by external excitatory input which, for simplicity, is assumed to be constant while the sensory object is present.

We consider a cluster of all-to-all coupled integrate and fire neurons. We average the firing rate of the network over many input presentations and analyze the shape of rise and decay of the overall firing rate. In each time step, the membrane potential *h_i_* of neuron *i* = 1, …, *N* receives additive external input *I_i_*(*t*) and excitation via recurrent connections with synaptic strength *w_i,j_* every time neuron *j* spikes, i.e., $n_{\text{sp}}^{j},$ see equation (1).

(1) $$h_{i,n+1}=h_{i,n}+I_{i,n}+\overset{N}{\underset{j=1}{\sum }}w_{i,j}\delta \left(n-n_{\text{sp}}^{j}\right)$$

where *δ*(*x*) = 0 for *x* ≠ 0 and *δ*(0) = 1. For continuous-time systems the time step becomes infinitesimally small and changes are expressed by a derivative d*h_i_*/d*t*. The dynamics can be described by a differential equation (2).

(2) $$\frac{\text{d}h_{i}}{\text{d}t}=I_{i}\left(t\right)+\overset{N}{\underset{j=1}{\sum }}w_{i,j}\delta \left(t-t_{\text{sp}}^{j}\right)$$

If *h_i_* reaches the firing threshold *θ* = 1, it delivers a spike to its postsynaptic neurons and is reset by the threshold value $h_{i}^{\text{post-spike}}=h_{i}^{\text{pre-spike}}-\theta .$ The external input *I_i_*(*t*) is drawn independently in each time step from a Gaussian distribution with a mean chosen such that a single neuron receives on average input equal to the difference of threshold and resting potential *θ* − *h*^0^. Without the recurrent coupling, a neuron would thus on average fire once during stimulus presentation.

**Figure 4:**
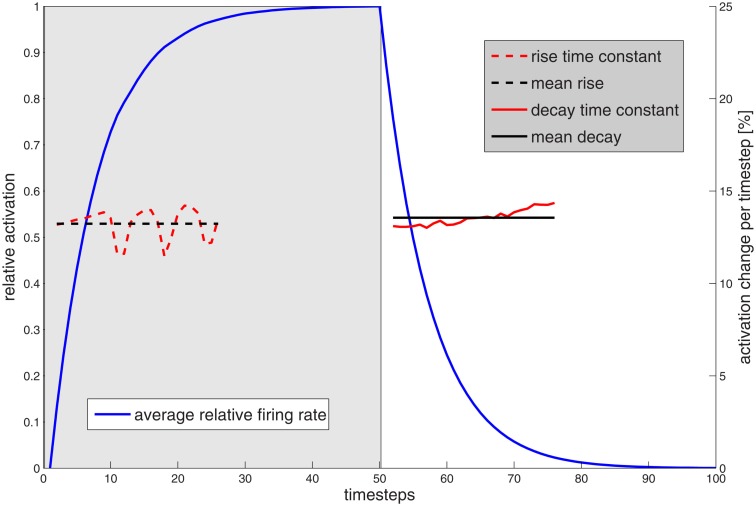
**Normalized average firing rate of the network as a response to input (applied from time step 0 to 50 indicated by the gray shaded region) and no input (blue)**. The firing rate is determined by binning the spikes in each time step. Normalization is performed by division by the average maximum firing rate at time 50. The fraction of two subsequent firing rates, which corresponds to the time constant in an exponential fixed-point dynamics, is shown in red. Black lines show the means of the respective red lines. The deviation of the blue curve from a purely exponential dynamics is apparent, but quite small, justifying the simplified dynamics as described in the text.

We simulated a network of *N* = 1000 neurons. A stimulus was shown for 1*s*, and the inter-stimulus interval was 1*s* (we are using 50 time steps per second). The total output of a neuron, i.e., the sum of all outgoing weights, was fixed to $\alpha =\sum_{i=1}^{N}w_{i,j}=0.87\;\forall j.$ The stochasticity of the input and the sensitivity of the network for fluctuations result in rather random single trial firing. However, on average a coherent behavior emerges. For the results shown in Figure 4, we averaged 10,000 trials to obtain a good estimation of the firing rate over time.

In order to derive a computationally simple dynamics for the representation variables of the GMNP, we are interested in the shape of the time course of rise and decay of the firing rate. A good candidate to describe the observed dynamics seems to be a set of coupled non-linear Langevin equations (Risken, 1996) of the basic form equation (3).

(3) $$\frac{\text{d}x}{\text{d}t}=h\left(x,t\right)+g\left(x,t\right)\Gamma \left(t\right)$$

The state of the system is ***x***, *t* is time, *h* is a function that describes drift forces that depend on the actual state and time and Γ(*t*) is a Gaussian diffusion term with zero mean 〈Γ(*t*)〉*_t_* = 0 and no correlation 〈Γ(*t*)Γ(*t*′)〉*_t_* = 2*δ*(*t* − *t*′).

Since theories of NP do not make any statements about noise influences, our strategy of aiming at a minimal model also suggests that we exclude noise effects in the model. The result is an exponential fixed-point dynamics with time constant τ.

$$\begin{array}{llll} x_{n+1} & =x_{n}+\tau \cdot \left(I-x_{n}\right) & & \text{(4)} \end{array}$$

$$\begin{array}{llll} \frac{\text{d}x}{\text{d}t} & =\tau \cdot \left(I-x\right) & & \text{(5)} \end{array}$$

In Figure 4 we show the averaged firing rate *f* and plot the relative change (*f_n+1_* − *f_n_*)/*f_n_* between two time steps in reference to the actual fixed-point, i.e., maximum firing rate 1 in case of input or 0 in the absence of input. The observed time constants are sufficiently constant to justify the simplified dynamics of equation (4) we used for the implementation of the GMNP.

**Figure 5:**
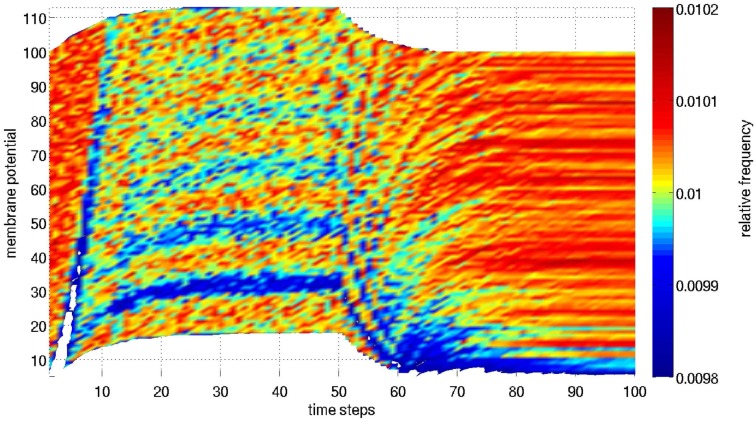
**Distribution of membrane potentials averaged over 10,000 trials**. Note that the potentials are mostly uniformly distributed, as the color map only covers values from 0.0098 to 0.0115. Nevertheless, the fine grained plot reveals the processes generating the firing rates analyzed in Figure 4: initially all neurons are pushed toward higher membrane potential by the input, leaving a relative gap that is propagated upwards. Then, assemblies of neurons that are characterized by increased membrane potentials form when the recurrent input builds up. Finally, the system relaxes and the less regular spikes rebuild a more equally distributed picture until no further spikes are generated.

The small periodicity of the rise time constant, even after averaging over a large number of runs, can be explained by the model structure. Figure 5 shows the distribution of membrane potentials averaged over 10,000 trials as shown in Figure 4. During input, all neurons are shifted in their membrane potential such that small potentials become improbable, to the benefit of superthreshold potentials. Most potential bins have a relative frequency of 0.0098 and 0.0115, which is near a uniform distribution. However, there is some structure that survives the averaging process. In the beginning, all units receive only external input. They are shifted upwards, leaving a gap which propagates through the entire range of potentials. Neurons that spiked are not reset to zero but lowered in their normalized potential by 1. Since they additionally receive recurrent as well as external input, virtually no neurons have membrane potentials between 0 and 0.15. As recurrent input tends toward a fixed-point, there is a trend of jumping into the band between 0.18 and 0.28 after spiking. This band is now shifted upwards by the same amount of activation. In every time step, a neuron jumps from one band to the next one. After the offset of input only decaying recurrent excitation is present.

#### 2.7.2 Feature variables

In the GMNP, all objects from input space are represented by tuples of feature activations. The number of relevant features can vary according to the paradigm. Information about a perceived object Ω is decomposed into its constituent features and then passed to the appropriate layers of the GMNP. Perceptual features drive feature detection variables of the system, whereas the information about the combination of all features to one object entity is governed by the binding layer. This defines the dynamic synaptic interaction between the feature variables of the object.

Feature variables $f_{i}^{j}$ represent whether a feature *i*, e.g., color, shape, or word shape, has the value *j*, e.g., green, etc. True information enters the system by the corresponding external input $F_{i}^{j}.$ The dynamics of a feature variable is determined by several driving forces that act simultaneously, see equation (6). The first one is an exponential drift toward $F_{i}^{j}.$ The time constant τ*_f_* of the drift equals either *ρ_f_* if the feature variable is lower than the input and rises by an active drive, or *δ_f_* if the input variable is lower than the current activation and the feature variable passively decays. $F_{i}^{j}$ is defined by constant unit input $\hat{F}$ in the presence of the respective feature in the display configuration. If the particular feature instance defines the object to be target or distractor, an additional input, excitatory or inhibitory, respectively, is applied to the corresponding feature variable. In case of feature perception, $F_{i}^{j}$ is set to a generic input strength $\hat{F}$ plus the current value of the variable accounting for the reception of input by only a subset of neurons in one assembly, similar to residual activations introduced in Schrobsdorff et al. (2007b). The residual overshoot of the input decays to the maximum input in the same way that would feature activation. In the case of feature absence, the input is set to the activation baseline value of $\overset{ˇ}{F},$ which is not necessarily zero.

(6) $$F_{i}^{j}=\left\{\;\begin{array}{cc} \;\hat{F}+f_{i}^{j} & \text{at display}\;\text{onset},\;\text{if}\;\text{instance}\;j\;\text{of}\;\text{feature}\;i\;\text{is}\;\text{present}\\ \;\delta_{f}\;\left(\hat{F}-F_{i}^{j}\right) & \text{during}\;\text{stimulus}\;\text{perception},\;\text{as}\;\text{long}\;\text{as}\;F_{i}^{j}>\hat{F}\\ \;\overset{ˇ}{F} & \text{at}\;\text{display}\;\text{offset}\\ \; \end{array}\right.$$

Both target selection mechanisms, target amplification and distractor inhibition add to the corresponding feature input ${\text{F}}_{i}^{j}$ resulting in the overall input $F_{i}^{j},$ see equation (7). Target amplification *A* is linearly increasing until a response is given and set to zero afterward, see equation (8). Distractor inhibition *I* is said to persist for some time, as it has to be retrenched after a response was given. Therefore, inhibition *I* increases linearly with slope *k* during perception and fades linearly after the decision was made, see equation (9).

$$\begin{array}{llll} {\text{F}}_{i}^{j} & =\left\{\begin{array}{cc} F_{i}^{j}+A & \text{if}\;\left\{i,j\right\}\;\text{defines}\;\text{the}\;\text{target}\\ F_{i}^{j}+I & \text{if}\;\left\{i,j\right\}\;\text{defines}\;\text{the}\;\text{distractor}\\ F_{i}^{j} & \text{otherwise} \end{array}\right. & & \text{(7)} \end{array}$$

$$\begin{array}{llll} \frac{\text{d}A}{\text{d}t} & =\alpha \;\text{during}\;\text{stimulus}\;\text{presentation} & & \text{(8)}\\ A & =0\;\text{no}\;\text{stimulus}\;\text{present} & & \end{array}$$

(9) $$\frac{\text{d}I}{\text{d}t}=\{\begin{array}{c} k\text{ during external input }\\ -k\text{ after the offset of input until }I=\text{0} \end{array}$$

The second term governing the dynamics of features is the loss of feature specificity in the absence of input defined by a broadening of activation with time constant *β*, within one feature toward the feature mean ${\left\langle f_{i}^{j}\right\rangle }_{i},$ without lowering the total activation of the respective feature layer. Additionally, feature activation is passed via existing bindings to the other feature instances belonging to the same object. If, e.g., the feature tuple {color, green}{shape, ball}{location, bottom} defining a green ball shown at the bottom of the visual scene is held by the binding variable *b*_{color, green}{shape, ball}{location, bottom}_, its value defines the amount of activation interchange between the variables $f_{\text{color}}^{\text{green}},$ $f_{\text{shape}}^{\text{ball}},$ and $f_{\text{location}}^{\text{bottom}}$ such that they all approach the object mean. There exists only one feature variable for green. Therefore multiple green objects experience a natural connection, as they share this variable. The last term that drives feature variables is the back projection of memorized episodes into the feature layer. Weighted by the matching value *r_k_* of the actual percept and the *k*th last memorized episode and the strength *e_k_* of the respective memory trace, the value of the feature variable at the respective response moment $e_{k}^{f_{i}^{j}}$ is fed back to the variable.

In total, the change of feature activation $f_{i}^{j}$ is the sum of four exponential drifts, given in equation (10). First, an adaptation toward input strength ${\text{F}}_{i}^{j}$ with time constant τ*_f_*. Second, an adaptation toward the mean of all activations in the particular feature layer ${\left\langle f_{i}^{j}\right\rangle }_{i}$ with time constant *β*. Third, an adaptation toward the mean of the other features of each object Ω the current feature belongs to with time constant *b*_Ω_, i.e., the current binding strength of that object. And finally, fourth, an adaptation toward the memorized value of the current variable $e_{k}^{f_{i}^{j}}$ with time constant *r_k_e_k_*, i.e., the product of the retrieval strength, the match between the percept and the *k*th memorized episode, and the current memory strength.

(10) $$\begin{array}{l} \frac{\text{d}f_{i}^{j}}{\text{d}t}=\tau_{f}\left(F_{i}^{j}-f_{i}^{j}\right)+\beta \left(\langle {f_{i}^{j}\rangle }_{i}-f_{i}^{j}\right)\\ \;+\sum_{\Omega ∍f_{i}^{j}}b_{\Omega }\left(\langle {f_{l}^{m}\rangle }_{f_{l}^{m}\in \Omega \backslash f_{i}^{j}}-f_{i}^{j}\right)+\sum_{k}r_{k}e_{k}\left(e_{k}^{f_{i}^{j}}-f_{i}^{j}\right) \end{array}$$

where

$$\tau_{f}=\{\begin{array}{c} \rho_{f}\text{ if }F_{i}^{j}>f_{i}^{j}\\ \delta_{f}\text{ if }F_{i}^{j}<f_{i}^{j} \end{array}$$

#### 2.7.3 Feature binding mechanism

The bindings are dynamic variables themselves that encode feature combinations within an object. Because the underlying structure (Schrobsdorff et al., 2007a) is a flexible but resource-constrained layer, the number of such binding variables is limited. When an object appears in stimulus space the feedback activation from the binding layer indicates whether the current object is already represented. This would correspond to an immediate recognition of the identity of the object. If the object is not yet represented, the weakest binding variable that is not subject to current input is overwritten, deleting the respective object from working memory. If an object is shown, the respective binding variable is driven with time constant *ρ_b_* toward a maximum strength $\hat{b}.$ If the percept of an object is gone, the respective binding variable passively decays with time constant *δ_b_* to zero, see equation (11).

(11) $$\frac{\text{d}b_{\left\{i_{k},j_{k}\right\}k}}{\text{d}t}=\left\{\begin{array}{cc} \rho_{b}\left(\hat{b}-b_{\left\{i_{k},j_{k}\right\}k}\right) & \text{if}\;\text{an}\;\text{object}\;\text{with}\;\text{the}\;\text{respective}\\ & \;\text{feature}\;\text{combination}\;\text{is}\;\text{perceived}\\ -\delta_{b}b_{\left\{i_{k},j_{k}\right\}k} & \text{if}\;\text{the}\;\text{percept}\;\text{is}\;\text{switched}\;\text{off} \end{array}\right.$$

If the binding slot is overwritten, we have *b*_{*ik, jk}k*_ = 0, i.e., object {*i_k_*, *j_k_*}*_k_* is not shown and is held by the weakest binding when a new display is uncovered containing a non-bound object {*i_l_*, *j_l_*}*_l_*.

#### 2.7.4 Short-term modulation of connectivity

The GMNP directs the information flow such that it achieves a decision whether a response will be computed anew from the perceptual input or will be retrieved from episodic memory. For this purpose, synaptic connections between the layers are either blocked or facilitated, depending on the old-new signal *o_k_* that is generated by comparing the *k*th last episode to the current percept. A blocking variable σ_block_ approaches *o_k_* with time constant τ_block_, see equation (13). The limiting value is set to 1, 1/2, or 0 depending on whether the signal is old, unclassified or new, respectively. This is applied if the model behavior is tuned to represent the temporal discrimination theory. The synaptic strength is scaled according to σ_block_ between a minimum synaptic strength ${\overset{ˇ}{\sigma }}_{f\to s}$ and an entirely open channel of σ*_f→*s*_* = 1, see equation (12).

(12) $$\sigma_{f\to s}=\left(1-{\overset{ˇ}{\sigma }}_{f\to s}\right)+{\overset{ˇ}{\sigma }}_{f\to s}\sigma_{\text{block}}$$

with

(13) $$\frac{\text{d}\sigma_{\text{block}}}{\text{d}t}=\tau_{\text{block}}\left(o_{k}-\sigma_{\text{block}}\right)$$

#### 2.7.5 Semantic variables

The role of the variables in the semantic layer is assigned by the central executive, depending on task demands. Therefore, a fixed description of the dynamics of semantic variables is not possible. We assume that after a hypothetical training phase that introduces a new task, the central executive has produced a reasonable gating function *S*(*f* ) of feature activations to the semantic layer. In the case of a naming paradigm this mapping can be as simple as the identity map from object shapes to semantic object category. The function *S*(*f* ) determines the fixed-point, which the semantic activation approaches at a rate *ρ_s_* or *δ_s_*, for an actively driven rise or a passive decay, respectively, see equation (15). Again the variables are subject to retrieval of former episodes analogous to feature variables. Additionally, the information flow is modulated by the connection factor σ*_f→*s*_*, see equation (14).

(14) $$\frac{\text{d}s^{j}}{\text{d}t}=\sigma_{f\to s}\tau_{s}\left(S^{j}\left(f\right)-s^{j}\right)+\underset{k}{\sum }r_{k}e_{k}\left(e_{k}^{s^{j}}-s^{j}\right)$$

where

(15) $$\tau_{s}=\left\{\begin{array}{cc} \rho_{s} & \text{if }\;S^{j}>s^{j}\\ \delta_{s} & \text{if }\;S^{j}<s^{j} \end{array}\right.$$

Actions of the GMNP are based on the most prominent activation of the semantic layer. We chose an adaptive-threshold mechanism to single out the highest activation. Only activations surpassing the threshold *s^θ^* are eligible to be passed on to the action layer.

#### 2.7.6 The adaptive-threshold in the semantic layer

As a decision mechanism for comparison tasks, the semantic layer is equipped with an adaptive-threshold *s^θ^*. The threshold variable itself obeys an exponential fixed-point dynamics on the basis of a scaled average of activation in the semantic layer. This is done similarly to the threshold behavior in Schrobsdorff et al. (2007b). The scaling of the average $\nu_{s^{\theta }}$ is dependent on the paradigm and should be set such that the fixed-point of the threshold is between the highest two semantic activations. As a consequence, the baseline activation $\overset{ˇ}{F}$ which is considered a virtual zero in the process has to be accounted for by only considering the difference to $\overset{ˇ}{F},$ see equation (16).

(16) $$\frac{1}{\tau_{s^{\theta }}}\frac{\text{d}s^{\theta }}{\text{d}t}=\nu_{s^{\theta }}\underset{j}{\sum }\left(s^{j}-\overset{ˇ}{F}\right)-\left(s^{\theta }-\overset{ˇ}{F}\right)$$

#### 2.7.7 Action representations

The action layer behaves similarly as the semantic layer, see equation (17). Action activation variables are driven toward an external input *A*(*s*, *f* ) that is computed from semantic and feature representations according to the task, i.e., given by a mapping function from the central executive. Depending on whether the adaptation is an actively driven rise or a passive decay, two respective time constants *ρ_a_*, *δ_a_* apply. An aspect that is easily overseen is the option not to respond, for example in cases where no target object is shown. This is represented by the formal action *a*^0^. *A^j^*(*s*, *f*, σ*_f,*s*→*a*_*) is designed such that whenever there is no target stimulus shown, e.g., between two trials, *A*^0^(*s*, *f*, σ*_f,*s*→*a*_*) equals 1. In case of stimuli triggering a response *A*^0^(*s*, *f*, σ*_f,*s*→*a*_*) equals 0. The variable σ*_f,*s*→*a*_* is the current synaptic strength between both feature and semantic layer toward the action layer.

(17) $$\frac{\text{d}a^{j}}{\text{d}t}=\tau_{a}\left(A^{j}\left(s,f,\sigma_{f,s\to a}\right)-a^{j}\right)+r_{a}\underset{k}{\sum }r_{k}e_{k}\left(e_{k}^{a^{j}}-a^{j}\right)$$

where

$$\tau_{a}=\left\{\begin{array}{cc} \rho_{a} & \text{if }\;A^{j}\left(s,f\right)>a^{j}\\ \delta_{a} & \text{if }\;A^{j}\left(s,f\right)<a^{j} \end{array}\right.$$

The relative retrieval of action representations *r_a_* is modulated contrary to the synaptic transmission to the action layer σ*_f,*s*→*a*_* reflecting the facilitation of action retrieval by an old-c an old episode which can be answered by retrieving a former response. Also, the modulation of information flow can decrease the retrieval of a response if a new episode is classified, see equation (18).

(18) $$r_{a}=\left(1\;+\;\text{max}\;\left({\overset{ˇ}{\sigma }}_{f,s\to a},{\overset{ˇ}{\sigma }}_{f\to s}\right)\;\right)\;-\;2\text{max}\;\left({\overset{ˇ}{\sigma }}_{f,s\to a},{\overset{ˇ}{\sigma }}_{f\to s}\right)\sigma_{\text{block}}$$

where

$$\sigma_{f,s\to a}=\left(1-{\overset{ˇ}{\sigma }}_{f,s\to a}\right)+{\overset{ˇ}{\sigma }}_{f,s\to a}\sigma_{\text{block}}$$

In order to model the decision making process in the action layer where a single action has to be chosen for execution, we introduce a threshold level analogous to the semantic layer described in Section 2.7.6, see equation (19). As input to the action layer ranges from 0 to 1, we do not have to care about baseline activation here.

(19) $$\frac{1}{\tau_{a^{\theta }}}\frac{\text{d}a^{\theta }}{\text{d}t}=\nu_{a^{\theta }}\underset{j}{\sum }a^{j}-a^{\theta }$$

Suprathreshold activations *a^j^* > *a^θ^* define the space of possible actions the system can take. If there is only one action that is suprathreshold, the corresponding action is executed. In case of *a*^0^ > *a^θ^*, the system does not do anything.

#### 2.7.8 Memory processes

Memory processes are modeled in a simple way. At points in time that mark the closure of an episode, in the present paradigm when an action has been performed, the entire state of the model is written down as one episode. The stored values are used to compute similarities between past episodes and a current percept, the retrieval strength *r_k_*. This similarity signal triggers an automatic retrieval of the former episodes. The greater the similarity, the stronger the memorized values drive the respective variables. Additionally, to account for memory decay with time, the presence of memorized episodes is set to a certain initial value $\hat{e}$ when the episode is written down, and then freely decays to zero with time constant *δ_e_*, see equation (20).

(20) $$\begin{array}{cc} \;e_{k}=\hat{e} & \text{if}\;\text{episode}\;k\;\text{is}\;\text{memorized}\\ \;\frac{\text{d}e_{k}}{\text{d}t}=-\delta_{e}e_{k} & \text{otherwise}\\ \; \end{array}$$

If a new episode is memorized, the *k*th last episode becomes the (*k* + 1)th last one, see equation (21).

(21) $$\left.\begin{array}{c} \;e_{k+1}^{v}=e_{k}^{v}\\ \;e_{1}^{v}=v\in \text{\{}f_{i}^{j},b_{{\text{\{}j_{k},i_{k}\text{\}}}_{k}},s^{j},a^{j}\text{\}}\\ \; \end{array}\right\}\;\text{when}\;\text{an}\;\text{action}\;\text{is}\;\text{taken}$$

To account for the classification, postulated, e.g., in temporal discrimination theory, we need a reliable old-new signal which is rather hard to get from only internal values, i.e., information that is accessible by the system itself. The current percept can only be assessed through the extracted feature. The intention is to have a value that is higher for a higher degree of similarity between the current percept and a memorized one. In other words, the difference of a current feature or binding value and the corresponding memory trace should be minimal, e.g. $\left(f_{i}^{j}-e_{k}^{f_{i}^{j}}\right).$ This is best achieved by the inverse of the sum of all differences. Still, there is a normalization problem, due to the varying stimulus displays. As the system is trained for the present task, it has some knowledge about the expected number of objects *n* in the display. However, the current objects can only be guessed by looking at the *n* strongest bindings. Therefore, we apply a normalization by the significance of a percept given by the sum over all currently perceived feature variables, divided by the number of features relevant to the task, see equation (22).

(22) $$r_{k}=\frac{\underset{i,j}{\sum }f_{i}^{j}}{{\#}_{f}}{\left(\underset{{\text{\{}i_{l},j_{l}\text{\}}}_{l}}{\sum }\left(\left|f_{i}^{j}-e_{k}^{f_{i}^{j}}\right|+\frac{1}{\hat{b}}\left|b_{{\text{\{}i_{l},j_{l}\text{\}}}_{l}}-e_{k}^{b_{{\text{\{}i_{l},j_{l}\text{\}}}_{l}}}\right|\right)\right)}^{-1}$$

where {*i_j_*, *j_l_*}*_l_* denotes a subjective percept, i.e., one of the objects being held by the *n* strongest bindings, *n* being the number of objects in one display.

#### 2.7.9 Connectivity modulation

Information gating is modeled by the dynamic opening or closing of synaptic transmissions between the different layers as well as the retrieval channel to the action layer. This modulation is governed by an old-new signal *o_k_* comparing the *k*th last episode to the current percept. The comparison process is modeled by locating the *k*th retrieval signal *r_k_* below, in between, or above a deviation *u* from a prototype time course for an intermediate resemblance of displays given by an exponential adaptation from an initial value $\overset{ˇ}{d}$ with time constant τ*_d_* toward a retrieval level $\overset{ˇ}{d}$ dividing old from new displays, see equation (23). In order to account for a greater uncertainty after the beginning of a trial, *u* shrinks exponentially with time constant τ*_u_*, see equation (24).

$$\begin{array}{llll} o_{k} & =\left\{\begin{array}{cc} 0 & \text{if}\;r_{k}>d+u\\ 1 & \text{if}\;r_{k}<d-u\\ \frac{1}{2} & \text{otherwise} \end{array}\right. & & \text{(23)} \end{array}$$

$$\begin{array}{llll} \frac{\text{d}u}{\text{d}t} & =-\tau_{u}u & & \text{(24)} \end{array}$$

where $d=\overset{ˇ}{d}$ and $u=\overset{ˇ}{u}$ at display onset, *d* = 0 and *u* = 0 at display offset, while the stimulus is present the following dynamics is observed, see equation (25).

(25) $$\frac{\text{d}d}{\text{d}t}=\tau_{d}\left(\hat{d}-d\right)$$

## 3 Results

Even though the most important aspect of the GMNP is the possibility to quantitatively compare different priming theories, the current contribution is not intended to establish the conditions and perform a thorough comparison, but the main result we are presenting is a framework which is general enough to quantify all theories of NP in a common language. Therefore, the current section is meant as a proof of concept to demonstrate the way the GMNP works.

### 3.1 Defining model parameters

In order to analyze the consequences of a theory, we define weights Ξ that switch on or off the effect of particular assumptions in a theory. These weights are meta-parameters insofar as they introduce constraints on the low-level parameters of the model that reflect the impact of a specific theoretical mechanism at a behavioral level. We label these variables according to the corresponding theory, see Table 3: Ξ_er_, episodic retrieval; Ξ_rr_, response retrieval; Ξ_ib_, inhibition vs. boost; Ξ_gt_, global threshold; Ξ_fsb_, feature-semantic block; Ξ_sab_, semantic action block; Ξ_td_, temporal discrimination.

**Table 3:** **Weights controlling the strength of the implementation of a theoretical account into the GMNP**.

	Model behavior for Ξ = 0	Model behavior for Ξ = 1
Ξ_er_	No retrieval at all	Maximum retrieval
Ξ_rr_	Only retrieval of response	Total retrieval
Ξ_ib_	Distractor inhibition	Target boost
Ξ_gt_	No activation interference	Forced decay and activation broadening
Ξ_fsb_	Full propagation	Retrieval blocks features semantic synapses
Ξ_sab_	Full propagation	Retrieval blocks semantic action synapses
Ξ_td_	Classical episodic retrieval	Old/new evaluation

*Their range is continuously between 0 and 1*.

Retrieval is controlled by adjusting the initial strength of a memory trace as it linearly determines the impact of retrieval. The modulation factor Ξ_er_ scales the maximum memory strength $\hat{e}.$ If Ξ_er_ is 0, no memory is written down, and therefore retrieval has no effect on the system behavior. If Ξ_er_ = 1, memories are stored initially with the maximum strength $\hat{e}$ and retrieval provides the input to the system described in Section 2.7.8.

The question whether the entire system state is retrieved or only the prime response, separates episodic retrieval from response-retrieval theory. These two assumptions are mutually exclusive. Therefore the weight Ξ_rr_ gradually shuts down the retrieval of activations in layers other than the action layer. If Ξ_rr_ = 1 the entire episode is retrieved, whereas, if Ξ_rr_ = 0, only the action layer receives memory input.

Distractor inhibition theory and the global threshold theory conflict with each other by either assuming inhibition of the distractor or a target boost, respectively. The weight Ξ_ib_ modulates input to the feature instance that identifies target and distractor. If Ξ_ib_ = 0, only the distractor receives inhibiting input, i.e., α = 0. If Ξ_ib_ = 1 only the target feature receives excitation, i.e., *k* = 0. Ξ_ib_ additionally adjusts the baseline activation level from 1/2 in the distractor inhibition case to 0 with target boost, where no sub-baseline activation is assumed.

At this point, a major gap in the retrieval accounts becomes obvious. They do not make any statements on what the direct computation of a trial may look like. The GMNP thus needs some decision making mechanism. In order to have the least effect of the decision making mechanism on priming effects in the case where we consider retrieval based mechanisms, we chose to have a pure target boost in the feature layers. Forced decay as well as activation broadening as inherent features of the global threshold theory will thus be controlled independently. Ξ_gt_ Linearly controls the broadening of activation *β* and the strength of the forced decay if two concepts compete for a feature instance.

Both temporal discrimination and episodic-retrieval theory postulate a decision of the system as to whether the current response should be generated directly from the input, or retrieved from memory. The corresponding modulation in the general model is done via the weight Ξ_fsb_. If Ξ_fsb_ = 0, there is a competition between direct computation and retrieval in the system. If Ξ_fsb_ = 1, the strength of retrieval, i.e., the similarity signal, triggers a shutdown of the synapses between features and semantic layer, modeling a decision of the system to only retrieve the response and drop the direct determination of the right answer.

In an excursion into episodic retrieval (Tipper and Cranston, 1985) argued in favor of blocking of the information flow in the episodic retrieval context right before the action selection state. This manifests in the general model as a blocking similar to Ξ_fsb_ described in the last paragraph. However, the block acts between the semantic and the action layer. The corresponding weight is Ξ_sab_.

A final weight is given by Ξ_td_ which controls the evaluation of a stimulus being old or new before retrieval is initiated. In the case Ξ_td_ = 0, the similarity signal determines the retrieval strength from the beginning of a trial, whereas if Ξ_td_ = 1 there is no retrieval unless the similarity signal surmounts the uncertainty region around the prototype similarity signal, as explained in Section 2.7.8.

Table 4 summarizes the values of the weights if the impact of a single theoretical account is to be evaluated. Note that some mechanisms are inherent to the GMNP such as activation propagation via the feature bindings. Therefore, these settings do not give a minimal computational model of the respective theory. Rather, we keep the unspecified mechanisms constant across all simulations.

**Table 4:** **Weight settings required by various theories**.

	Ξ_er_	Ξ_rr_	Ξ_ib_	Ξ_gt_	Ξ_fsb_	Ξ_sab_	Ξ_td_
Distractor inhibition	0	0	0	0	0	0	0
Global threshold	0	0	1	1	0	0	0
Episodic retrieval	1	1	1	0	0	0	0
Response retrieval	1	0	1	0	0	0	0
Temporal discrimination	1	1	1	0	1	1	1

### 3.2 Voicekey paradigm

**Figure 6:**
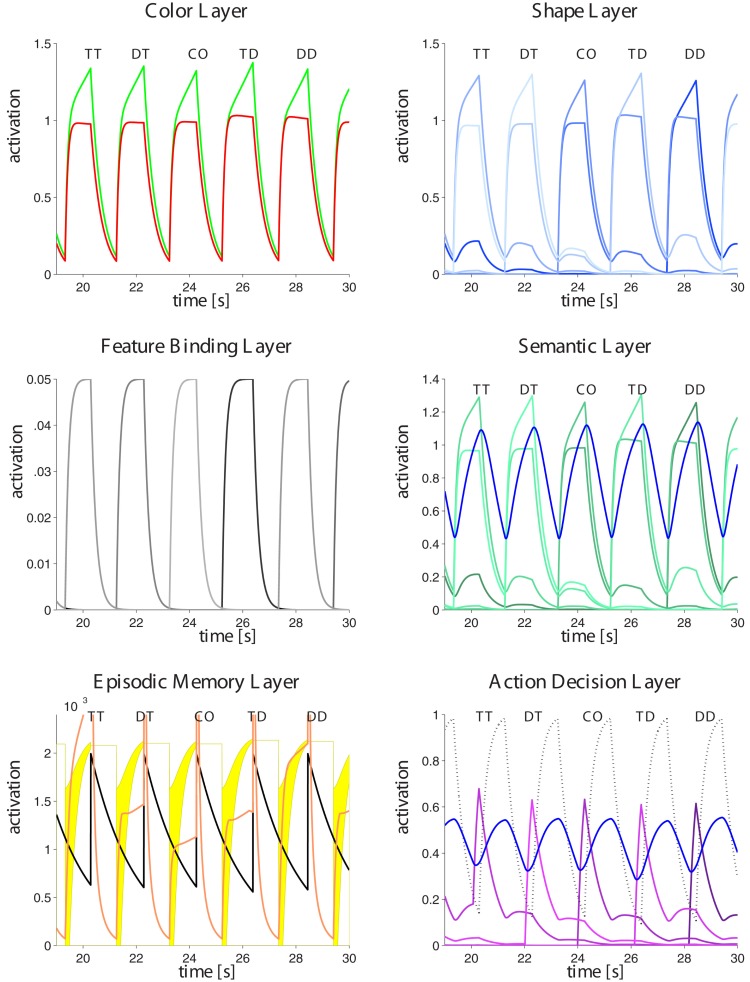
**Activation traces over time in the different layers of the GMNP in the voicekey paradigm described in Section 2.2**. Different colors correspond to different variables in the respective layer. A few traces are to be highlighted: solid blue lines in both the semantic and the action layer correspond to the respective threshold variable, black in the episodic memory layer denotes the strength of the memory trace, yellow is the uncertainty region for the old-new signal which is drawn in orange. The model is in classical episodic-retrieval mode, see Section 3.1. Targets are boosted and the entire episode retrieved. Retrieval is apparent in the plots by the re-rise of formerly active variables.

The following section will show an example of the GMNP in a voicekey paradigm, see Section 2.2. To show the internal dynamics of the GMNP, all relevant variables are plotted over nine trials including all five conditions in Figure 6. The weights are tuned to episodic retrieval, i.e., there are no activation interferences in the feature layers. In response to the perceptual input, the target color green is boosted and activation exchanged via the bindings. In addition, activation is retrieved from memory.

The presentation of a red and a green pictogram drives the two color and the two shape representations in the respective layers. The central executive delivers additional input to green which augments the activity of the target object’s shape via the bindings. The semantic representations are fed by a one-to-one mapping from the shape layer, i.e., S(f)=𝕀. The plot of the episodic memory layer shows the memory strength in black which decays with time from a fixed value at memory initialization which takes place at the point a response is given. In orange, the plot shows the similarity signal which linearly modulates the retrieval of a former trial. The signal is highest for the TT trial, intermediate for DT, TD, and DD in ascending order. In the action layer, the black dotted trace is for the no-action response, see Section 2.7.7. The selection of the target in the semantic layer, i.e., the object surpassing the semantic threshold, is fed forward to the action layer.

The present simulation was run with the following values of the relevant parameters: Ξ_er_ = 1, Ξ_rr_ = 1, Ξ_ib_ = 1, Ξ_gt_ = 0, Ξ_fsb_ = 0, Ξ_sab_ = 0, Ξ_td_ = 0, α = 0.0005, $\overset{ˇ}{F}$ = 1, *t*_recognition_ = 50, *t*_afterimage_ = 30, *t*_motor_ = 80, *ρ_f_* = 0.01, *δ_f_* = 0.003, $\hat{b}=0.05,$ #*_b_* = 7, *ρ_b_* = 0.008, *δ_b_* = 0.005, $\tau_{s^{\theta }}=0.002,$ $\nu_{s^{\theta }}=0.51,$ *ρ_a_* = 0.004, *δ_a_* = 0.002, $\tau_{a^{\theta }}=0.002,$ $\nu_{a^{\theta }}=0.5,$ $\hat{e}=0.002,$ *δ_e_* = 0.003.

Negative priming in DT trials and positive priming in TT trials are with 24 and 53 ms at rather realistic scales (see Table 5). The present example together with three other realizations is part of the GMNP-software bundle.

**Table 5:** **Mean reaction time and effect strength for the priming conditions CO, DT, TT produced by the GMNP in episodic-retrieval mode as described in Section 3.2**.

	〈RT〉 [ms] (SD)	Effect [ms]
CO	976 (7)	–
DT	1000 (10)	−24
TT	923 (22)	53
TD	1134 (11)	−73
DD	1049 (9)	−158

### 3.3 Analysis of the word-picture paradigm

As a showcase example of how to exploit the capabilities of the GMNP to gain more insight in the interaction of the different processes that are involved in NP, we now present a detailed analysis of the GMNP when faced with a word-picture comparison task as it is described in Ihrke et al. (2011). This particular paradigm has a second factor besides priming condition, which is response repetition. Therefore, the labels of the experimental conditions receive an additional suffix, i.e., **s** for response switch and **r** for response repetition. By a parallel implementation, we are able to perform a gradient descent on the parameter set, while keeping the theory semaphores adjusted to each of the settings described in Table 4. Thereby, we obtain information about which of the theoretical assumptions implemented in the GMNP is able to reproduce the experimental results to which degree. Although we optimized the model for the DT and TT conditions, we provide the results for the other conditions that were present in the corresponding experiment as well, which can be regarded as parameter-free predictions. These predictions are there to provide the reader with an idea of how the model can inform further experimental work.

After convergence, the root mean squared error between experimental and simulated effects and control reaction time of the GMNP instance set to distractor inhibition behavior is the lowest (see Table 6). The obtained parameters in that case are: Ξ_er_ = Ξ_rr_ = Ξ_ib_ = Ξ_gt_ = Ξ_fsb_ = Ξ_sab_ = Ξ_td_ = 0, iota = 0.000001, *β* = 0.00155, ϕ = 0.00011, α = 0.0005, $\overset{ˇ}{F}$ = 1, *t*_recognition_ = 50, *t*_afterimage_ = 30, *t*_motor_ = 80, *ρ_f_* = 0.009, *δ_f_* = 0.003, $\hat{b}=0.05,$ #*_b_* = 7, *ρ_b_* = 0.0096, *δ_b_* = 0.005, $\tau_{s^{\theta }}=0.002,$ $\nu_{s^{\theta }}=0.4131,$ σ_shape→*s*_ = 0.1, σ_word→*s*_ = 0.12, σ*_s_→*a** = 1, *ρ_a_* = 0.0036, *δ_a_* = 0.002, $\tau_{a^{\theta }}=0.002,$ $\nu_{a^{\theta }}=0.6,$ $\hat{e}=0.002,$ *δ_e_* = 0.003.

**Table 6:** **Root mean squared error (RMSE) after a converged gradient descent fit to the absolute reaction time of a control trial (COs and COr) and the priming effects of DTs, DTr, and TTs and TTr while keeping the theory weights fixed**.

	RMSE
Distractor inhibition	14.0
Temporal discrimination	22.5
Episodic retrieval	34.6
Response retrieval	38.1
Global threshold	39.1

**Figure 7:**
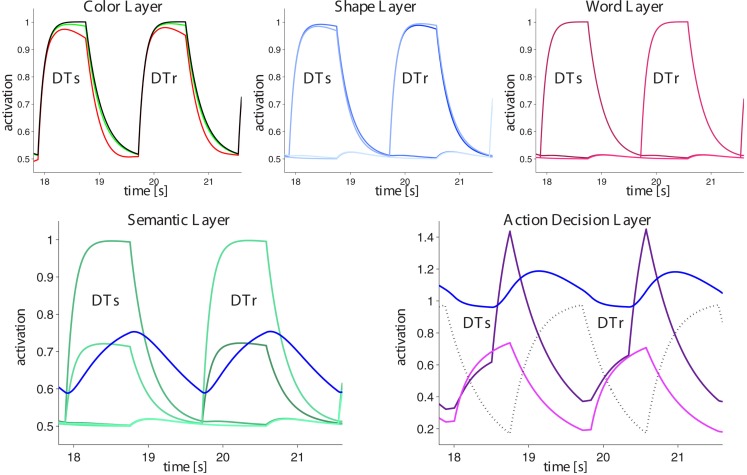
**Activation traces over time in the relevant layers of the GMNP in the comparison paradigm**. For coloring see Figure 6. The model is tuned to distractor inhibition mode, see Section 3.1. Two different conditions are shown: DTs, the former target becomes the current target and the reaction switches (from no to yes in this case); and DTr, again the former distractor becomes the current target but now the reaction does not switch (yes in both prime and probe trial). This plot illustrates the difficulty of comparing theories that are developed in a different context. Distractor inhibition theory itself is not able to explain a reaction time difference between the two conditions, as it is only formulated on a semantic level. Indeed GMNP does not show a difference in the traces except in the action layer, where persistent activation and relative inhibition causes the observed effects.

The corresponding reaction times, given in Table 7, show a very good reproduction. The interaction between response relation and priming condition gave rise to response-retrieval theory, as distractor inhibition theory *per se* is not able to explain it, although it is remarkable that distractor inhibition, as it is implemented in the GMNP, seems to best explain the experimental data. There are several aspects to discuss in that context. First, the GMNP does not reduce to the original implementation of distractor inhibition theory with one on- and one off-cell, controlling recognition of objects. The framework of the GMNP, i.e., its layer structure, the feature decomposition, and the dedicated action layer offer a flexibility that the original theory did not have. Second, the inability of the GMNP in distractor inhibition mode to perfectly fit both DTs and DTr simultaneously may point to the limitations of a pure inhibitory account and toward the necessity of retrieval mechanisms to fully explain the interaction as postulated in Rothermund et al. (2005), for a graphical comparison of DTs and DTr trials see Figure 7.

**Table 7:** **Simulated reaction times and effects by the GMNP in distractor inhibition mode compared to experimental results from Ihrke et al. (2011), after fitting model parameters to minimize the RMSE in control RT and the effect sizes for TT and DT conditions**.

	GMNP RT [ms]	Experimental RT [ms]
COs	825.5	821.2
DTs	829.8	842.0
TTs	840.4	835.8
TDs	830.5	814.9
TTs	819.8	817.6
COr	835.5	838.4
DTr	826.3	829.5
TTr	814.3	816.7
TDr	815.4	840.7
DDr	836.2	824.4
**EFFECTS**		
DTs	−4.2	−20.8
TTs	−14.8	−14.6
TDs	−5.0	6.3
DDs	5.7	3.6
DTr	9.1	8.9
TTr	21.2	21.7
TDr	20.1	−2.3
DDr	−0.7	14.0

When encountering apparent contradictions to the original formulation of a theory, another great advantage of computational modeling becomes important: it is very easy to extract detailed information about the conditions that are responsible for unattended behavior, thus providing quick and definite explanations for it. In the described example it seems like distractor inhibition theory is not well implemented in the GMNP as the corresponding setting produces the best fit for an interaction of response relation and priming condition, one of the known weak points of distractor inhibition as it cannot explain these results. But when examining the behavior of the GMNP in detail, the effect is solely present in the action layer, which has not been taken into account by the original distractor inhibition theory. The RMSE between DTs and DTr is less than a tenth of the difference in the action layer when averaged over one trial. Further, this numerical experiment shows that the postulate that response repetition interaction with priming is incompatible with distractor inhibition seems too strict. Obviously, adding a response mechanism with slowly decaying response activation is sufficient to enable a distractor inhibition model to show such an interaction, even if it is admittedly imperfect.

## 4 Discussion

Combining experimental evidence from behavioral experiments with basic system neuroscientific mechanisms, we present a GMNP that incorporates all presently relevant theories of the phenomenon. The model clearly identifies differences of experimental conditions and is thus able to resolve existing inconsistencies among the important theories. The model is tested in a number of standard scenarios and is shown to be easily extendable to non-standard versions of priming experiments.

The GMNP gives a unified framework to quantify each of the theories for NP, allowing, for the first time, a quantitative comparison of the impact of the proposed mechanisms. The identification of weights for the different accounts makes it convenient to compare the different predictions in a particular setting.

Negative priming presents itself as a complex phenomenon which has been accounted for by different theoretical descriptions focusing on specific experimental paradigms. A computational theory can provide a comprehensive framework under these conditions if it is both sufficiently abstract and flexible to reveal similarity and to describe the differences between the aspects of the phenomenon under consideration. Interestingly, the adaptation of the computational model by means of weights (see Table 4) gives a straightforward recipe for generating predictions. In principle there are 2^7^ = 128 possible configurations for the values of the weights, only five of which related to experimental and theoretical studies investigated so far in the current literature. Obviously not all configurations are interesting or even meaningful, but a few more studies can be easily suggested that would provide insight into the necessity of the model’s components while so far we can only judge whether they are sufficient.

The simulated reaction times in Section 3.2 and the other examples featured in the provided code, show that the behavior of the GMNP is far from being robust against even small parameter changes. Even though a stable model is much more convenient from a theoretical point of view, we consider this instability necessary in order to account for the multitude of different findings in connection with NP. However, we have to face the question of whether the model is able to fit any pattern of experimentally recorded data with just the right parameter settings. Due to the high dimensionality of the parameter space and the sensitivity of the GMNP, this question cannot be answered conclusively by the means of parameter scanning techniques. In fact, an important next step for the GMNP is parameter reduction by determining as many values as possible by comparisons with trusted experimental results, e.g., for the availability of afterimages, decay times of feature bindings, etc. The detail of the GMNP is also easily capable of showing partial reaction times as described in Ihrke et al. (2012) and Schrobsdorff et al. (2012). Therefore, a good way to limit the range of the parameter space would be to have a series of time-marker experiments specially designed to reveal processing stages that are measurable in the GMNP. Till that time the GMNP can only be a basis on which a concrete discussion on the nature of NP theories and paradigms can be made.

Besides the direct computation of reaction times, the structure of GMNP allows for numerical fitting via a multitude of algorithms. As an example we showed a gradient descent search for an optimal parameter set, keeping the theory weights fixed in order to compare the different theories in terms of flexibility to fit a given set of experimental results. Although a pure gradient descent may not be suitable for such a complex and huge parameter space, the numerical experiments in Section 3.3 already showed a surprising result: expanding the distractor inhibition model by only a reaction mechanism with a threshold and persistent activation as well as relative inhibition, provides a context which is able to produce the interaction of response relation and priming condition, which is otherwise considered to be the weakest point of distractor inhibition theory.

Another promising extension follows from the abstract formulation of relations among mechanisms that are involved in NP. Just as NP theories are formulated using concepts such as memory or central executive which are borrowed from other areas in psychology, the computational implementation of relations among these concepts also has a wider applicability than NP. The main components of the GMNP qualify it already as a *cognitive architecture* similar, e.g., to ACT-R (Anderson et al., 1997) or SOAR (Laird et al., 1987). Beyond this, it would be interesting to discuss the ensuing perspectives for design of artificial cognitive systems, such as for the control of an autonomous robot.

## Conflict of Interest Statement

The authors declare that the research was conducted in the absence of any commercial or financial relationships that could be construed as a potential conflict of interest.

## Footnotes

^1^The source code containing several paradigm examples is available through the project web site http://www.bccn-goettingen.de/projects/gmnp
